# Effects of different embryo culture media on birthweight following assisted reproductive technology

**DOI:** 10.1093/hropen/hoaf041

**Published:** 2025-07-09

**Authors:** Ming Li, Zhengyang Zhao, Qingqing Tao, Jin Huang, Ying Lian, Yue Li, Shengli Lin, Ping Liu, Qin Li, Rong Li, Jie Qiao

**Affiliations:** Department of Obstetrics and Gynecology, Center for Reproductive Medicine, School of Public Health and State Key Laboratory of Female Fertility Promotion, Peking University Third Hospital, Beijing, China; National Clinical Research Center for Obstetrics and Gynecology, Peking University Third Hospital, Beijing, China; Key Laboratory of Assisted Reproduction, Ministry of Education, Peking University, Beijing, China; Beijing Key Laboratory of Reproductive Endocrinology and Assisted Reproductive Technology, Beijing, China; Department of Obstetrics and Gynecology, Center for Reproductive Medicine, School of Public Health and State Key Laboratory of Female Fertility Promotion, Peking University Third Hospital, Beijing, China; Department of Obstetrics and Gynecology, Center for Reproductive Medicine, School of Public Health and State Key Laboratory of Female Fertility Promotion, Peking University Third Hospital, Beijing, China; Department of Obstetrics and Gynecology, Center for Reproductive Medicine, School of Public Health and State Key Laboratory of Female Fertility Promotion, Peking University Third Hospital, Beijing, China; National Clinical Research Center for Obstetrics and Gynecology, Peking University Third Hospital, Beijing, China; Key Laboratory of Assisted Reproduction, Ministry of Education, Peking University, Beijing, China; Beijing Key Laboratory of Reproductive Endocrinology and Assisted Reproductive Technology, Beijing, China; Department of Obstetrics and Gynecology, Center for Reproductive Medicine, School of Public Health and State Key Laboratory of Female Fertility Promotion, Peking University Third Hospital, Beijing, China; National Clinical Research Center for Obstetrics and Gynecology, Peking University Third Hospital, Beijing, China; Key Laboratory of Assisted Reproduction, Ministry of Education, Peking University, Beijing, China; Beijing Key Laboratory of Reproductive Endocrinology and Assisted Reproductive Technology, Beijing, China; Department of Obstetrics and Gynecology, Center for Reproductive Medicine, School of Public Health and State Key Laboratory of Female Fertility Promotion, Peking University Third Hospital, Beijing, China; National Clinical Research Center for Obstetrics and Gynecology, Peking University Third Hospital, Beijing, China; Key Laboratory of Assisted Reproduction, Ministry of Education, Peking University, Beijing, China; Beijing Key Laboratory of Reproductive Endocrinology and Assisted Reproductive Technology, Beijing, China; Department of Obstetrics and Gynecology, Center for Reproductive Medicine, School of Public Health and State Key Laboratory of Female Fertility Promotion, Peking University Third Hospital, Beijing, China; National Clinical Research Center for Obstetrics and Gynecology, Peking University Third Hospital, Beijing, China; Key Laboratory of Assisted Reproduction, Ministry of Education, Peking University, Beijing, China; Beijing Key Laboratory of Reproductive Endocrinology and Assisted Reproductive Technology, Beijing, China; Department of Obstetrics and Gynecology, Center for Reproductive Medicine, School of Public Health and State Key Laboratory of Female Fertility Promotion, Peking University Third Hospital, Beijing, China; National Clinical Research Center for Obstetrics and Gynecology, Peking University Third Hospital, Beijing, China; Key Laboratory of Assisted Reproduction, Ministry of Education, Peking University, Beijing, China; Beijing Key Laboratory of Reproductive Endocrinology and Assisted Reproductive Technology, Beijing, China; Department of Obstetrics and Gynecology, Center for Reproductive Medicine, School of Public Health and State Key Laboratory of Female Fertility Promotion, Peking University Third Hospital, Beijing, China; National Clinical Research Center for Obstetrics and Gynecology, Peking University Third Hospital, Beijing, China; Key Laboratory of Assisted Reproduction, Ministry of Education, Peking University, Beijing, China; Beijing Key Laboratory of Reproductive Endocrinology and Assisted Reproductive Technology, Beijing, China; Department of Obstetrics and Gynecology, Center for Reproductive Medicine, School of Public Health and State Key Laboratory of Female Fertility Promotion, Peking University Third Hospital, Beijing, China; National Clinical Research Center for Obstetrics and Gynecology, Peking University Third Hospital, Beijing, China; Key Laboratory of Assisted Reproduction, Ministry of Education, Peking University, Beijing, China; Beijing Key Laboratory of Reproductive Endocrinology and Assisted Reproductive Technology, Beijing, China; Department of Obstetrics and Gynecology, Center for Reproductive Medicine, School of Public Health and State Key Laboratory of Female Fertility Promotion, Peking University Third Hospital, Beijing, China; National Clinical Research Center for Obstetrics and Gynecology, Peking University Third Hospital, Beijing, China; Key Laboratory of Assisted Reproduction, Ministry of Education, Peking University, Beijing, China; Beijing Key Laboratory of Reproductive Endocrinology and Assisted Reproductive Technology, Beijing, China

**Keywords:** IVF, embryo culture medium, birthweight, large for gestational age, macrosomia

## Abstract

**STUDY QUESTION:**

Does the type of embryo culture medium affect the birthweight of newborns conceived by ART?

**SUMMARY ANSWER:**

After fresh embryo transfers, singleton newborns in the G5 and HTF groups exhibited higher birthweight *z*-scores and increased risks of being large for gestational age (LGA) compared to those in the Cook group.

**WHAT IS KNOWN ALREADY:**

Current studies have not yet determined whether embryo culture medium affects birthweight and, if such an effect does exist, the significance of embryo culture medium among all the influencing factors is not yet clear.

**STUDY DESIGN, SIZE, DURATION:**

A retrospective cohort study including 23 403 fresh ET cycles between 1 January 2010 and 31 December 2022 at the reproductive medical center of a university-affiliated hospital was conducted.

**PARTICIPANTS/MATERIALS, SETTING, METHODS:**

We performed a retrospective cohort study including 23 403 fresh ET cycles. Four embryo culture media were analyzed: Cook, G5-PLUS, G5, and HTF. Multiple linear regression analysis was used to evaluate potential associations between embryo culture medium and birthweight *z*-score. Logistic regression analysis was used to evaluate potential associations between embryo culture medium and the risk of LGA and macrosomia. Random forest models were constructed to conduct significance analysis of all factors that may affect birthweight *z*-score.

**MAIN RESULTS AND THE ROLE OF CHANCE:**

The cohort comprised 4453, 8460, 7463, and 3027 singletons in the Cook, G5-PLUS, G5, and HTF groups, respectively. Compared to the Cook group, newborns in the G5 and HTF groups had higher birthweight *z*-scores (increased by 0.069 units, *P *< 0.001, and 0.073 units, *P = *0.002, respectively) and higher risks of LGA (OR: 1.25, 95% CI: 1.12–1.39, *P *< 0.001; OR: 1.20, 95% CI: 1.05–1.37, *P *= 0.009, respectively), while newborns in the G5 group also had a higher risk of macrosomia (OR: 1.21, 95% CI: 1.06–1.39, *P* = 0.006). Of the main factors influencing birthweight, the embryo culture medium had a moderate significance.

**LIMITATIONS, REASONS FOR CAUTION:**

Due to commercial and regulatory reasons, various culture media were used for different periods, thus resulting in the different number of cases for the four culture media groups.

**WIDER IMPLICATIONS OF THE FINDINGS:**

Our analysis revealed that the type of embryo culture medium directly affected birthweight *z*-scores and the risk of LGA and macrosomia in newborns conceived by ART. Consequently, the selection of embryo culture medium should be made cautiously. In addition, there’s also a need for more post-market data on culture media for embryology labs.

**STUDY FUNDING/COMPETING INTEREST(S):**

This study was supported by National Key Research and Development Program of China (2023YFC2705604 and 2024YFC2706900) and the National Natural Science Foundation of China (82071721, 82371706, and 82288102). All authors have no conflicts of interest to report.

**TRIAL REGISTRATION NUMBER:**

N/A.

WHAT DOES THIS MEAN FOR PATIENTS? This study looked at whether the type of liquid used to grow embryos in the laboratory (i.e. embryo culture medium) affects the baby’s birthweight after IVF or similar treatments.

Birthweight is an important factor in neonatal health, and previous studies have not determined whether the embryo culture medium has a direct influence. We analyzed data from over 23 000 treatment cycles comparing four common embryo culture media (Cook, G5-PLUS, G5, and HTF). Compared to embryos cultured in Cook medium, those cultured in G5 or HTF medium produced newborns of significantly higher birthweight and a greater incidence of being born large for gestational age (i.e. birthweight >90th percentile of weight for newborns of the same gestational age and sex). Newborns from the G5 group also had a higher risk of macrosomia (birthweight >4000 g).

These findings indicate that the choice of embryo culture medium may influence newborn size. Clinicians and patients should consider this factor when selecting laboratory materials for fertility treatment. It is also essential to conduct post-market monitoring of embryo culture media to ensure their safety and efficacy in clinical practice.

## Introduction

Infertility is defined as the failure to establish a clinical pregnancy after 12 months of regular and unprotected sexual intercourse (Vander Borght and Wyns, 2018) and has become a significant population problem that is estimated to affect between 8% and 12% of couples of reproductive age worldwide (Inhorn and Patrizio, 2015). ART is the main form of infertility treatment at present, and is applied widely across the world (Farquhar and Marjoribanks, 2018; Saha *et al.*, 2021). In 2018, more than 10 million children were born by ART worldwide, and it is responsible for 7.9% of all children born in Europe and up to 5.1% of all children born in the USA (Pinborg *et al.*, 2023). However, substantial evidence suggests that children conceived by ART are at increased risks of adverse outcomes compared to those conceived naturally (Centers for Disease Control and Prevention (CDC), 2009; Pandey *et al.*, 2012; Dhalwani *et al.*, 2016; Liberman *et al.*, 2017; Lei *et al.*, 2019; Reig and Seli, 2019; Wennerholm and Bergh, 2020; Wang *et al.*, 2022; Graham *et al.*, 2023; Pinborg *et al.*, 2023). Consequently, it is essential that we investigate the potential risks of ART, focusing particularly on the association of ART with unhealthy birthweight (Reig and Seli, 2019).

The most significant difference between an ART pregnancy and a natural pregnancy is that, in an ART pregnancy, the zygote and embryo need to develop in a culture medium for a specific period of time. An embryo culture medium is used throughout the entire IVF-embryo transfer (IVF-ET) process to provide a specific environment for nutrition, survival, and development for the gametes and embryos, and is therefore essential for embryo development and newborn health (Chronopoulou and Harper, 2015). The composition of the embryo culture media provided by different manufacturers can differ markedly, and these differences may exert complex effects on embryo development and the health outcomes of newborns (Morbeck *et al.*, 2014a). For example, a multi-center RCT study found that singletons in the Vitrolife G5 group had a significantly lower birthweight *z*-score when compared to the HTF group (Kleijkers *et al.*, 2016). Conversely, another national study in the UK found no significant association between the embryo culture medium and either birthweight or the birthweight *z*-score (Castillo *et al.*, 2020a). Other studies have investigated the risk of large for gestational age (LGA) and macrosomia, but the results have been inconsistent (Dumoulin *et al.*, 2010; Nelissen *et al.*, 2012; Vergouw *et al.*, 2012; Carrasco *et al.*, 2013; Lemmen *et al.*, 2014; Wunder *et al.*, 2014; Zhu *et al.*, 2014; Yin *et al.*, 2015; Gu *et al.*, 2016; Kleijkers *et al.*, 2016; Sacha *et al.*, 2022; Zheng *et al.*, 2022; Chen *et al.*, 2023). Insufficient sample sizes, some covariates, and variations in embryo culture medium composition and laboratory factors may have contributed to the lack of consistent conclusions in previous studies, and therefore more research evidence is needed on the specific effects of different embryo culture media on birthweight and as well as the risk of LGA and macrosomia.

Another interesting question relating to embryo culture medium is whether human serum albumin (HSA) should be pre-mixed. In the early stages of ART, HSA was mixed manually by the operator prior to use. However, over recent years, manufacturers have tended to produce ready-to-use embryo culture medium and HSA is added at the time of manufacture. HSA contain a range of potentially bioactive molecules and contaminants (Morbeck *et al.*, 2014b). In addition, the two mixing methods differ in accuracy in terms of concentration and dosage of HSA, and may also lead to differences in shelf life and degree of mixing of the culture medium. Few studies have investigated the effects of ready-to-use embryo culture medium on birthweight. Our literature searches identified only one previous study which reported that ready-to-use embryo culture medium may exert impact on birthweight *z*-scores, but failed to provide a mechanistic explanation for this finding (Zhu *et al.*, 2014). However, the sample size of this previous study was small and only two types of embryo culture media were analyzed. Whether ready-to-use embryo culture medium will also affect birthweight and the risk of LGA and macrosomia remains unclear.

To answer these important questions, we conducted a retrospective cohort study involving 23 403 women who underwent IVF or ICSI treatment at the Reproductive Medicine Center of Peking University Third Hospital, with fresh ET cycles. We compared the effects of four commonly used embryo culture media on the birthweight *z*-score as well as the risk of LGA and macrosomia. Our research aimed to further elucidate the relationship between embryo culture medium and the birthweight of newborns conceived by ART.

## Materials and methods

The Science Research Ethics Committee of the Peking University Third Hospital Medical, Beijing, China, approved this retrospective cohort study. The committee decided that the study was exempt from human participant research review and that informed consent could be waived because this study involved secondary data analysis without personally identifiable information. Our study conforms to the Strengthening the Reporting of Observational Studies in Epidemiology (STROBE) reporting guidelines for cohort studies (reference: IRB00006761-M2024280).

### Study design and population

**Figure 1.**
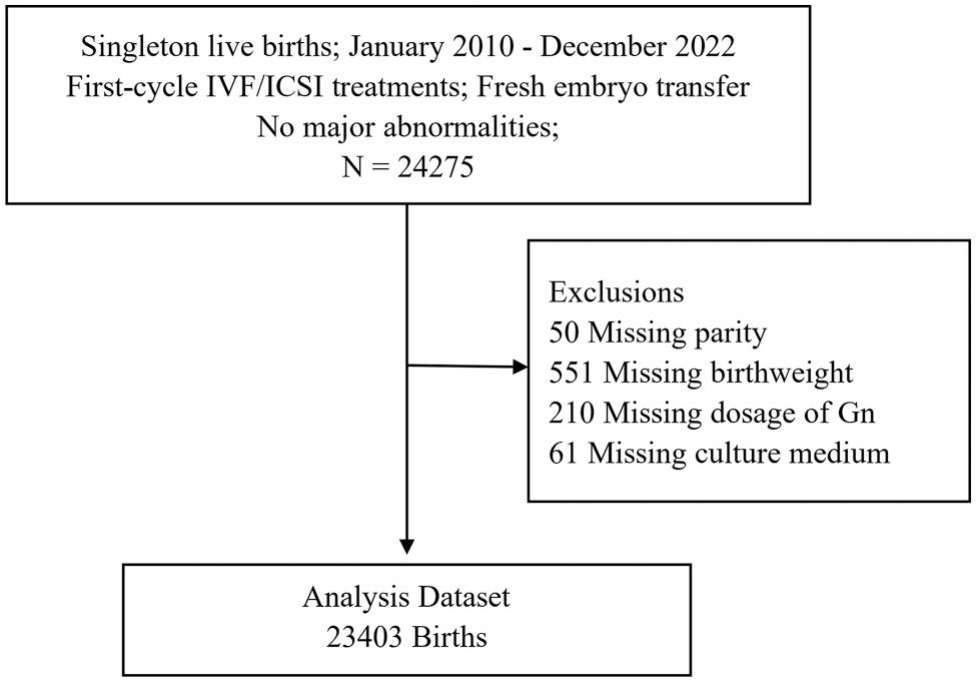
**Flowchart showing patient recruitment.** Gn, gonadotropin.

This was a retrospective cohort study. All women who underwent IVF or ICSI via fresh ET cycles and delivered a singleton live birth without major congenital malformations at the Reproductive Medical Centre of Peking University Third Hospital between 1 January 2010 and 31 December 2022 were included in our study cohort. All women had achieved successful fertilization and embryo development resulting in clinical pregnancies, but not all of them delivered at this hospital. Information relating to patients and treatment cycles was downloaded from the electronic medical record system, including: patient characteristics (age, parity, BMI), type of infertility (primary or secondary infertility), causes of female infertility, ovarian stimulation protocol, dosage of gonadotropin (Gn), frozen sperm, sperm source, fertilization method (IVF or ICSI), ET stage (cleavage or blastocyst stage), the number of embryos transferred, and common obstetric complications (including gestational hypertension, preterm birth, and vanishing twin syndrome). The professional staff at the center collected the newborn characteristics via telephone follow-up and recorded these data into the electronic medical record system, also including gender, gestational age (GA), and birthweight. We included only first-cycle IVF/ICSI treatments and excluded cycles that were missing basic information. Ultimately, a total of 23 403 IVF/ICSI cycles were included in this study (Fig. 1).

### Laboratory protocols

The following four commercially available embryo culture media were used: Cook (Cook, Sydney, Australia); G5™-PLUS (Vitrolife, Gothenburg, Sweden); G5™ (Vitrolife); and HTF (Lonza, Verviers, Belgium). Of these, Cook and G5™-PLUS were ready-to-use media and included 5 mg/ml of purified HSA. G5™ and HTF require artificial supplementation with 5 mg/ml of purified HSA prior to use: HSA solution™ (Vitrolife) for G5™ and HSA solution (IVF Online, Toronto, Canada) for HTF. Mineral oil was obtained from Vitrolife. Oocytes were retrieved and placed in a humidified incubator at 37°C, 5% or 6% CO_2_ (5% for HTF and 6% for Cook, G5™-PLUS and G5™). For IVF, oocytes were inseminated 3–4 h after oocyte retrieval. For ICSI, the cumulus cells were stripped 2 h after oocytes retrieval, and ICSI was performed as previously described (Palermo *et al.*, 1992). Fertilization examination and embryo culture were performed according to the laboratory’s routine procedures as previously described (Li *et al.*, 2021). The zygotes and embryos were also cultured in a 5% or 6% CO_2_ humidified incubator at 37°C. In this study, Cook media was used from 2011 to 2022, HTF was used from 2010 to 2017, and both G5-PLUS and G5 were used throughout the study period, so the number of women using the different culture media was not exactly the same. In all four groups, the embryo culture media was rotated approximately once every 3 days without any particular selection, and when the supply was insufficient, we continued to rotate the existing stock to maintain continuity.

### Outcomes

The primary outcome of this study was the birthweight *z*-score: (Observed birthweight−Mean birthweight of the reference population stratified by GA and sex)/(SD of the reference population stratified by GA and sex); which inherently adjusts for GA and sex. This metric can accurately evaluate the development of newborn children. We used a calculation tool provided by the INTERGROWTH-21st project (a multicenter study establishing international standards for newborn size) to calculate the *z*-score (http://intergrowth21.ndog.ox.ac.uk/). There have been many studies that have proven the reliability of the INTERGROWTH-21st project (Roggero *et al.*, 2024; Relph *et al.*, 2025). The use of birthweight *z*-score as an outcome allows for a more accurate assessment of the biological impact of embryo culture medium on fetal growth. The secondary outcomes of this study were LGA and macrosomia. Based on previous studies, we defined LGA as newborns with a birthweight exceeding the 90th centile (Nyadanu *et al.*, 2023). Fetal macrosomia was defined as a birthweight exceeding 4000 g, regardless of the GA or sex of the newborn (Poupon *et al.*, 2020).

### Statistical analysis

Cycles were grouped according to the different types of embryo culture media. Multiple linear regression analysis was used to evaluate potential associations between embryo culture medium and birthweight *z*-score with other potential confounding factors, including female age, female BMI, parity, type of infertility, causes of female infertility, ovarian stimulation protocols, dosage of Gn, frozen sperm, sperm source, fertilization method (IVF or ICSI), ET stage (cleavage or blastocyst stage), and the number of embryos transferred. We calculated the general variance inflation factor (VIF) for all independent variables in the multiple linear regression model to understand the multicollinearity between the variables and to ensure the reliability of the model (Kim, 2019). Logistic regression analysis was used to evaluate potential associations between embryo culture medium and the risk of LGA and macrosomia with the same potential confounders as in the multiple linear regression model. The odds ratios (ORs) and 95% CIs of each group of media compared with the Cook group were calculated, respectively. To evaluate the significance of embryo culture medium among all factors that could influence birthweight, we constructed random forest models to conduct significance analysis of all factors that may affect birthweight *z*-score. These models use the %IncMSE (Percentage Increase in Mean Squared Error) to measure how much a variable’s removal deteriorates the model’s predictive performance. Specifically, for each variable, we calculated the MSE of the full model and the MSE of a model excluding that variable. %IncMSE = [(Full Model MSE − Reduced Model MSE) / Full Model MSE] × 100%. A higher %IncMSE indicates greater variable importance, and removing it may cause a larger drop in model accuracy. We also performed sensitivity analysis by excluding ET stage, the type of infertility, or female BMI from the model, respectively. Also, since both G5 and G5-PLUS were made by the Vitrolife company and have the same core ingredients, with the only difference being the timing of HSA addition, we combined these two groups into one group, the Vitrolife group, in one of the sensitivity analysis. This grouping aligns with their functional equivalence in clinical protocols, ensuring comparability while isolating the impact of HSA supplementation timing. The sensitivity analysis approach was determined based on previous relevant studies (Nelissen *et al.*, 2012; Zhu *et al.*, 2014; Castillo *et al.*, 2020b). All statistical analyses were performed with R software, version 4.2.3 (R Core Team, 2021). A *P*-value <0.05 for a two-sided test was considered statistically significant.

## Results

### Patients, cycles, and neonatal characteristics

A total of 23 403 cycles were analyzed, with 4453, 8460, 7463, and 3027 women and their singleton live newborns in the Cook group, G5-PLUS group, G5 group, and HTF group, respectively. Patients, cycles, and neonatal characteristics are shown in Table 1. The mean female age was 32.2 ± 4.07 years in the Cook group, 32.4 ± 4.09 years in the G5-PLUS group, 31.9 ± 4.17 years in the G5 group, and 32.0 ± 4.27 years in the HTF group; with significant differences between groups in terms of patient age (*P *< 0.001). The mean female BMI (kg/m^2^) was 22.7 ± 3.63 in the Cook group, 22.6 ± 3.57 in the G5-PLUS group, 22.5 ± 3.40 in the G5 group, and 22.5 ± 3.36 in the HTF group; there were also significant differences between the groups in terms of BMI (*P *= 0.004). We also found that significant differences existed between the four groups in terms of the type of infertility, causes of female infertility, fertilization method, ET stage, the number of embryos transferred, and the incidence of gestational hypertension (*P *< 0.001 for all). Newborn gender distribution and GA did not differ significantly when compared between the four groups (*P *= 0.929 and *P *= 0.946, respectively). However, we detected a significant difference between the four groups in terms of birthweight *z*-score (*P *< 0.001) and observed that the ready-to-use culture media (Cook and G5-PLUS) appeared to be associated with a lower birthweight *z*-scores. We also detected significant differences in the proportions of LGA and macrosomia between the four groups (*P *< 0.001 and *P *= 0.006, respectively), and that ready-to-use media appeared to be associated with a lower proportions of LGA and macrosomia cases.

**Table 1.** Characteristics of the patients, IVF/ICSI cycles, and neonatal characteristics^a^.

	Cook (N = 4453)	G5-PLUS (N = 8460)	G5 (N = 7463)	HTF (N = 3027)	*P*-value
**Maternal characteristics**					
Age, mean (SD), year	32.2 ± 4.07	32.4 ± 4.09	31.9 ± 4.17	32.0 ± 4.27	<0.001*^b^
Primipara	4187 (94.0)	7900 (93.4)	7032 (94.2)	2846 (94.0)	0.142
BMI, mean (SD), kg/m^2^	22.7 ± 3.63	22.6 ± 3.57	22.5 ± 3.40	22.5 ± 3.46	0.004*
**Type of infertility**					<0.001*
Primary	2660 (59.7)	4898 (57.9)	4098 (54.9)	1643 (54.3)	
Secondary	1793 (40.3)	3562 (42.1)	3365 (45.1)	1384 (45.7)	
**Causes of female infertility**					<0.001*
Tubal and pelvic factors	1399 (31.4)	2996 (35.4)	3130 (41.9)	1330 (43.9)	
Ovary	1144 (25.7)	1995 (23.6)	1253 (16.8)	479 (15.8)	
Endometriosis	241 (5.4)	480 (5.7)	361 (4.8)	127 (4.2)	
Unexplained	112 (2.5)	205 (2.4)	103 (1.4)	35 (1.2)	
Other	1557 (35.0)	2784 (32.9)	2616 (35.1)	1056 (34.9)	
**Fertilization method**					<0.001*
IVF	2701 (60.7)	5253 (62.1)	4530 (60.7)	1592 (52.6)	
ICSI	1752 (39.3)	3207 (37.9)	2933 (39.3)	1435 (47.4)	
**Embryo transfer stage**					<0.001*
Cleavage	4190 (94.1)	8097 (95.7)	7124 (95.5)	2913 (96.2)	
Blastocyst	263 (5.9)	363 (4.3)	339 (4.5)	114 (3.8)	
**Number of embryos transferred**	1.87 ± 0.35	1.92 ± 0.38	1.99 ± 0.39	2.08 ± 0.44	<0.001*
**Gestational hypertension**	69 (1.5)	177 (2.1)	73 (1.0)	31 (1.0)	<0.001*
**Preterm birth**	263 (5.9)	504 (6.0)	445 (6.0)	185 (6.1)	0.986
**Vanishing twin syndrome**	189 (4.2)	385 (4.6)	356 (4.8)	149 (4.9)	0.470
**Newborn gender**					0.929
Female	2103 (47.2)	4015 (47.5)	3514 (47.1)	1445 (47.7)	
Male	2350 (52.8)	4445 (52.5)	3949 (52.9)	1582 (52.3)	
**Gestational age, mean (SD), week**	38.7 ± 1.36	38.7 ± 1.35	38.7 ± 1.36	38.7 ± 1.35	0.946
**Birthweight, mean (SD), g**	3310 ± 473	3320 ± 470	3350 ± 478	3350 ± 459	<0.001*
**Birthweight *z*-score, mean (SD)**	0.243 ± 0.969	0.257 ± 0.977	0.323 ± 1.000	0.333 ± 0.978	<0.001*
**Large for gestational age (>90th percentile)**	619 (13.9)	1241 (14.7)	1258 (16.9)	498 (16.5)	<0.001*
**Macrosomia (>4000 g)**	352 (7.9)	688 (8.1)	707 (9.5)	262 (8.7)	0.006*

a Unless otherwise indicated, data are expressed as number (percentage). Percentages have been rounded and may not total 100.

b Chi-square tests or one-way ANOVA.

* *P* < 0.05.

### Multiple linear regression analysis and logistic regression analysis

**Figure 2.**
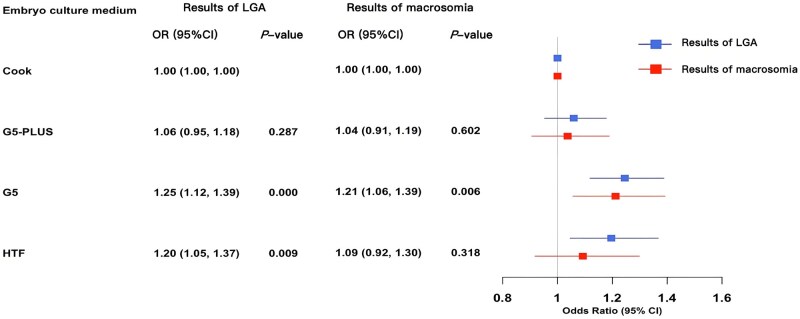
**The relationship between embryo culture medium and the risk of LGA or macrosomia.** LGA, large for gestational age; OR, odds ratio.

The relationship between birthweight *z*-score and embryo culture medium is shown in Table 2. Compared with the Cook group, the birthweight *z*-score of newborns in the G5 group increased by an average of 0.069 units (*P *< 0.001) while the birthweight *z*-score of newborns in the HTF group increased by 0.073 units on average (*P *= 0.002) after controlling for potential influencing factors. The VIF for all variables was <1.5, indicating no significant multicollinearity. The results of multiple linear regression analysis, including all covariables, are presented in Supplementary Table S1. The relationship between the risks of LGA and macrosomia and the embryo culture medium is shown in Fig. 2. Using the Cook group as a reference, the risk of LGA was significantly higher in the G5 group (OR: 1.25; 95% CI: 1.12–1.39, *P *< 0.001) and the HTF group (OR: 1.20; 95% CI: 1.05–1.37, *P *= 0.009). In addition, the risk of macrosomia was significantly higher in the G5 group (OR: 1.21; 95% CI: 1.06–1.39, *P *= 0.006). In our subgroup analysis, which stratified the dataset by the type of infertility, newborns in the G5 group were associated with a significantly higher birthweight *z*-score in the primary infertility group (Supplementary Table S2). Furthermore, newborns in the G5, G5-PLUS, and HTF groups all had significantly higher risks of LGA or macrosomia compared to those in the Cook group (Supplementary Table S3).

**Table 2.** Multiple linear regression analysis of the effect of embryo culture medium on birthweight *z*-score.

	β^a^	*P*-value
**Medium (vs Cook)**		
Medium 1 (G5-PLUS)	0.009	0.597
Medium 2 (G5)	0.069	<0.001*^b^
Medium 3 (HTF)	0.073	0.002*

a β represents the regression coefficient.

b two-sided tests.

* *P* < 0.05.

### Evaluation of the importance of various characteristics for birthweight

**Figure 3.**
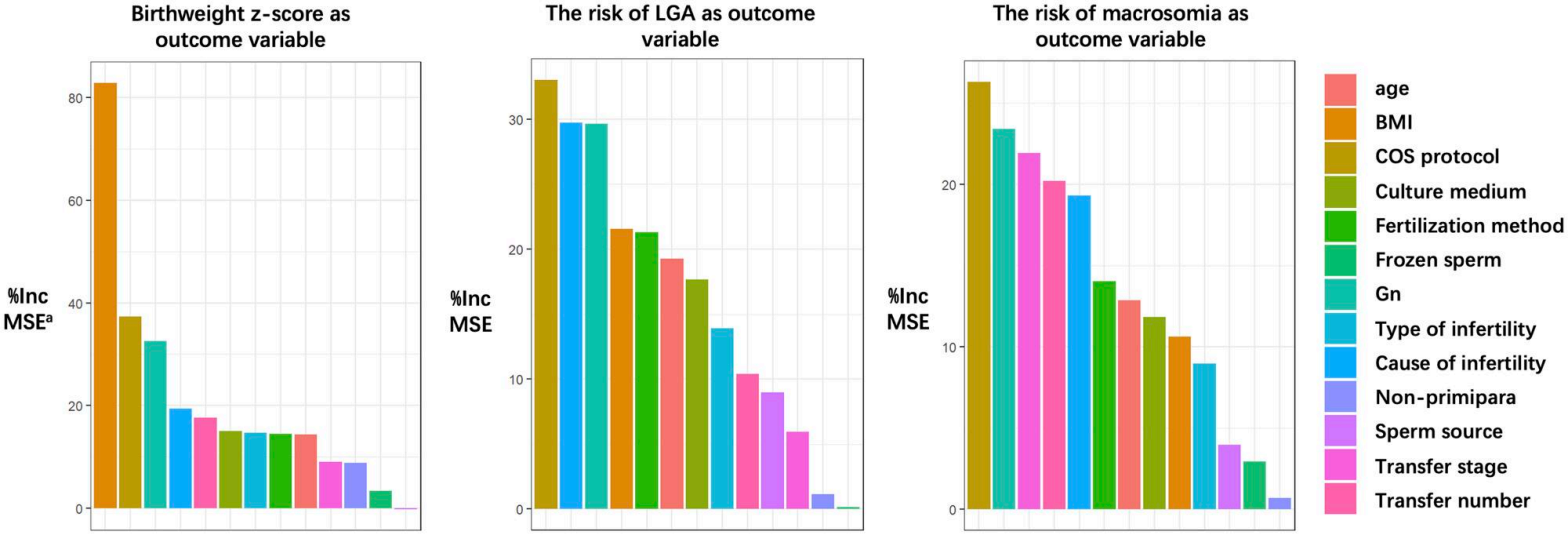
**Significance rankings of variables by random forest models.**  ^a^%IncMSE (Increase in Mean Squared Error) represents the mean reduction of model accuracy after removing a specific variable. The greater the %IncMSE, the greater the significance of the variable as an influencing factor. COS, ovarian stimulation; Gn, gonadotrophin.

Next, we constructed random forest models to evaluate the importance of all features that may affect birthweight *z*-scores and the risk of LGA and macrosomia. The outcomes of our analysis are shown in Fig. 3. Female BMI was identified as the most important feature for birthweight, and the importance of the embryo culture medium was similar to that of female age. This suggests that embryo culture medium has a relatively high predictive importance for birthweight *z*-score in the model, and combined with the results of the multiple linear regression model, we conclude that embryo culture medium is a significant factor influencing birthweight.

### Sensitivity analysis

When excluding ET stage, the type of infertility, or female BMI from the model, respectively, the associations between embryo culture medium and birthweight *z*-score or the risk of LGA and macrosomia remained consistent with the results of the original models (Supplementary Tables S4–S9). Specifically, newborns in the G5 and HTF groups had relatively higher birthweight *z*-score and a higher risk of LGA compared to those in the Cook group, and newborns in the G5 group also had a higher risk of macrosomia. Similar conclusions were evident when combining cycles using the two Vitrolife media (G5 and G5-PLUS), referred to as the Vitrolife group. The Vitrolife and HTF groups were associated with a significantly higher birthweight *z*-scores and risks of LGA than the Cook group (Supplementary Tables S10 and S11). These results indicated that the results of the multiple linear regression model were robust.

## Discussion

In this single-center birth cohort study, we found that the type of embryo culture medium had an impact on birthweight *z*-score, and the risk of LGA and macrosomia, in newborns conceived by ART. Of the four commonly used embryo culture media tested in this study, we found that the birthweight *z*-scores for newborns in the G5 and HTF groups were significantly higher than those in the Cook group. Newborns in the G5 and HTF groups also had higher risks of LGA and those in the G5 groups also had higher risks of macrosomia than those in the Cook group. In addition, in the random forest models, the impact of embryo culture medium on these outcomes was similar in importance to that of female age, suggesting that the predictive importance of embryo culture medium on *z*-score is relatively high. Therefore, we believe that the embryo culture medium is a significant factor affecting birthweight and the risk of LGA and macrosomia, and its selection should be considered more carefully in ART.

The importance of changes in birthweight cannot be ignored (Barker, 2007), as substantial evidence clear demonstrates that birthweight affects health in adulthood (Barker, 2007; Kerkhof and Hokken-Koelega, 2012; Kapral *et al.*, 2018; Mohseni *et al.*, 2020; Johnsson *et al.*, 2022). Therefore, understanding the effects of embryo culture medium on birthweight is of significant importance. However, the findings reported in the existing literature are inconsistent. Two initial studies, published in 2010 and 2012 and involving fewer than 300 singletons, reported that the type of embryo culture medium affected birthweight (Dumoulin *et al.*, 2010; Nelissen *et al.*, 2012). Subsequently, Hassani *et al.* (2013) and Eskild *et al.* (2013) also reported an association between the type of embryo culture medium and birthweight, with sample sizes of 174 and 2435, respectively. Conversely, other studies, with a sample size ranging from ∼1600 to 3000, concluded that the type of embryo culture medium was not associated with birthweight (Carrasco *et al.*, 2013; Lin *et al.*, 2013; Gu *et al.*, 2016). In a recent study, Castillo *et al.* (2020a) collected information from 18 693 ART newborns and found that embryo culture medium was not statistically associated with unadjusted birthweight or gestation-adjusted birthweight. Another large study, which included 7588 newborns reached a similar conclusion. However, these researchers noted that due to clinical and chronological factors, the effect of embryo culture medium on birthweight could not be completely ruled out (Castillo *et al.*, 2020b).

In our study, we detected a significant association between the type of embryo culture medium and birthweight *z*-score; these findings were inconsistent with most of the findings reported previously. However, previous studies suggesting that embryo culture medium did not have an effect on birthweight may have been limited by small sample sizes, a lack of important clinical information, and the inability to rule out laboratory factors. In the present study, we used a birth cohort with a larger sample size, more detailed data, and more stable culture conditions. Furthermore, all embryo culture media used during the same period were rotated approximately once every 3 days without any particular selection, which enhanced the reliability of our conclusion. Although there may be limitations to the clinical significance of a slight increase in individual birthweight *z*-score, the increasing number of children currently being born through ART, even with only a small increase in birthweight per child, will in the future result in a significant increase in the societal burden of associated long-term diseases.

The effects of embryo culture medium on the risk of LGA and macrosomia are more concerning, as numerous studies have shown that LGA and macrosomia can lead to adverse health outcomes in adulthood, including obesity, cardiovascular disease, diabetes (Carolan-Olah *et al.*, 2015; Derraik *et al.*, 2020; Suhag *et al.*, 2022), and mental health conditions (Van Lieshout and Boyle, 2011; Van Lieshout *et al.*, 2020). However, there is no definitive consensus on this issue. Most previous studies have reported that embryo culture medium does not affect the risk of LGA and macrosomia (Dumoulin *et al.*, 2010; Nelissen *et al.*, 2012; Vergouw *et al.*, 2012; Lemmen *et al.*, 2014; Wunder *et al.*, 2014; Yin *et al.*, 2015; Gu *et al.*, 2016; Kleijkers *et al.*, 2016; Sacha *et al.*, 2022; Chen *et al.*, 2023), even though some of these studies identified an effect on birthweight (Dumoulin *et al.*, 2010; Nelissen *et al.*, 2012; Kleijkers *et al.*, 2016). Only studies by Zheng *et al.* (2022) and Zhu *et al.* (2014) identified a link between embryo culture medium and the risk of LGA and macrosomia in newborns. In the present study, we found that the type of embryo culture medium can exert significant effects on the risk of LGA and macrosomia. The increased risk of LGA and macrosomia clearly demonstrates the importance of considering the impact on the health of the offspring when selecting embryo culture medium, beyond just the success rates of ART treatment.

An increasing number of IVF centers are increasingly inclined to use ready-to-use culture medium. Notably, our study found that ready-to-use culture medium appears to be associated with lower birthweight *z*-scores and a reduced incidence of LGA and macrosomia. One possible reason may be the different composition of HSA used in each culture medium. Another reason may be that ready-to-use culture medium may have a more accurate concentration of HSA, and have received more efficient mixing with HSA, thus eliminating deviations in concentration of HSA and the problems that occur following inadequate mixing of HSA when preparations are generated manually. A third potential explanation may be the difference in the duration of time from mixing (the culture medium and serum) to its application (as a culture medium). A culture medium containing manually incorporated HSA is used immediately after mixing (within about 1 h); thus, the duration of time from mixing to application is short. In contrast, the duration of time from mixing to application for ready-to-use media is longer because the serum and culture medium are mixed together usually more than 1 week before application and are mixed in more thorough manner over a greater space of time. Therefore, serum and culture medium can be more thoroughly mixed to exert the positive effects of serum, such as more fully chelating toxins, so it affects neonatal outcomes. As is well known, HSA is an important component of the culture medium, having a dramatic impact of embryo development (Swain, 2014; Swain *et al.*, 2016; Martínez-Casado *et al.*, 2024). On one hand, HSA has been found to act as a surfactant, stabilize membranes, modulate the microenvironment, chelate toxins, and provide a source of nitrogen, all of which are beneficial to embryo development. On the other hand, HSA has been reported to be a source of ammonium and to include stabilizers required for protein solutions, such as octanoic acid, that may be embryotoxic. Different culture media use their own serum, which has different compositions and concentrations, and there are differences in the process of using serum (ready-to-use or not), and thus, HSA is a large source of potential variation for the culture system. However, since the impact of these factors remains speculative, it is currently unclear as to whether ready-to-use embryo culture medium (specifically, its unique pre-mixed HSA configuration) can influence the birthweight of newborns and what the possible mechanisms involved maybe. Further research is now needed to clarify these issues.

Commercially available embryo culture media are associated with certain safety problems. The exact formula of these media is often not fully disclosed, and in many countries, culture media are considered as medical devices rather than drugs. This classification implies that clinical safety trials may be lacking (Chronopoulou and Harper, 2015). We advocate for the more careful application of these media in ART. Regulators should hold manufacturers to stricter standards, requiring them to publish full details of their formulations and to conduct more rigorous clinical safety tests, similar to those used for drugs, before the embryo culture medium is marketed.

The advantage of our study lies in addressing the gaps associated with previous literature, at least to some extent. Furthermore, our sample size is larger than most previous studies. All of our cases were recruited from the same laboratory, thus effectively eliminating the influence of laboratory factors. Four different culture media were compared at the same time in order to investigate the effects of culture media on birthweight more comprehensively, and more importantly, all embryo culture media used during the same period were rotated approximately once every 3 days without any particular selection. Moreover, we constructed random forest models to obtain a ranking of the significance of the independent variables, so that we could more intuitively understand the contribution of the embryo culture medium among all factors affecting the birthweight and the risk of LGA and macrosomia.

Our research also has some limitations that need to be considered. First, there were differences in the number of cases between different medium groups. Due to commercial and regulatory reasons, various culture media were used for different time periods, thus resulting in the different number of cases for the four culture medium groups. In addition, since this study mainly focused on embryo culture medium and the main conclusions were based on multiple linear regression model, other variables (e.g. ovarian stimulation protocols, dosage of Gn) that ranked high in the random forest models were not explained in detail. Among them, the results of the multiple linear regression model showed that different ovarian stimulation protocols significantly affected birthweight, which was consistent with previous studies (Jwa *et al.*, 2019). However, the effect size of dosage of Gn in the multiple linear regression model was close to zero and not statistically significant (*P *> 0.05), and therefore, it was concluded that this variable lacked a statistically significant independent linear association with *z*-score. The effects of these important variables on birthweight still need further in-depth study. Finally, this study utilized a retrospective design, which may introduce selection bias and confounding interference. However, since all information was collected and recorded in real time during prior medical procedures, there was no recall bias in this study. This mimics, to some extent, the characteristics of a prospective study. In addition, the large sample size of this study and the fact that the culture media were not selected according to patient condition or operator preference, but were kept in a 3-day rotation, somewhat compensated for the shortcomings of the retrospective study design.

## Conclusions

In this study, we found that the type of embryo culture medium can affect the birthweight *z*-scores and the risks of LGA and macrosomia of newborns conceived by ART, and that its importance is similar to that of maternal age, and cannot be ignored. The selection and use of embryo culture medium should be more scientific and cautious in the ART process. Furthermore, before an embryo culture medium is marketed, manufacturers need to carry out a more rigorous and scientific safety assessment with reference to the relevant standards of drugs, and it is also essential to conduct post-market monitoring of embryo culture media to ensure their safety and efficacy in clinical practice.

## Supplementary Material

hoaf041_Supplementary_Data

## Data Availability

The data underlying this article will be shared on reasonable request to the corresponding author.
