# Supplementary material for: Effects of different embryo culture media on birthweight following assisted reproductive technology
Source: Hum Reprod Open. 2025 Jul 9;2025(3):hoaf041. doi: 10.1093/hropen/hoaf041 (PMC12296354; doi:10.1093/hropen/hoaf041)
Supplement: hoaf041_Supplementary_Data [file hoaf041_supplementary_data.docx]

**Supplementary Data**

**Supplementary Table S1**. Complete multiple linear regression results with all covariables.

|  | Estimate | Std. Error | t value | *P*-value |
| --- | --- | --- | --- | --- |
| (Intercept) | -0.526 | 0.092 | -5.741 | <0.001* ^a^ |
| Female age | -0.003 | 0.002 | -1.788 | 0.074 |
| Parity |  |  |  |  |
| 1, as reference |  |  |  |  |
| 0 | -0.147 | 0.028 | -5.255 | <0.001* |
| Female BMI | 0.048 | 0.002 | 26.123 | <0.001* |
| Type of infertility |  |  |  |  |
| Primary, as reference |  |  |  |  |
| Secondary | 0.055 | 0.014 | 3.935 | <0.001* |
| Causes of female infertility |  |  |  |  |
| Tubal and pelvic factors, as reference |  |  |  |  |
| Ovary factors | -0.044 | 0.018 | -2.415 | 0.016* |
| Endometriosis | -0.043 | 0.030 | -1.427 | 0.154 |
| Others | -0.044 | 0.016 | -2.694 | 0.007* |
| COS (Controlled Ovarian Stimulation) protocols |  |  |  |  |
| Long and very long protocols, as reference |  |  |  |  |
| Spontaneous ovulation | -0.267 | 0.342 | -0.779 | 0.436 |
| Short protocol | 0.132 | 0.030 | 4.448 | <0.001* |
| Very short protocol | -0.266 | 0.118 | -2.252 | 0.024* |
| Antagonist protocol | -0.028 | 0.015 | -1.905 | 0.057 |
| Microstimulus protocol | 0.087 | 0.121 | 0.724 | 0.469 |
| Others | -0.131 | 0.129 | -1.012 | 0.311 |
| Dosage of gonadotropin (Gn) | 0.000 | 0.000 | 0.704 | 0.482 |
| Frozen sperm |  |  |  |  |
| Yes, as reference |  |  |  |  |
| No | -0.107 | 0.041 | -2.608 | 0.009* |
| Sperm source |  |  |  |  |
| Surgical semen extraction, as reference |  |  |  |  |
| Natural ejaculation | -0.010 | 0.028 | -0.343 | 0.732 |
| Fertilization method |  |  |  |  |
| ICSI, as reference |  |  |  |  |
| IVF | -0.020 | 0.015 | -1.396 | 0.163 |
| Embryo transfer stage |  |  |  |  |
| Cleavage, as reference |  |  |  |  |
| Blastocyst | 0.156 | 0.033 | 4.776 | <0.001* |
| Number of embryos transferred | 0.024 | 0.018 | 1.349 | 0.177 |
| Embryo culture medium |  |  |  |  |
| Cook, as reference |  |  |  |  |
| G5-PLUS | 0.009 | 0.018 | 0.528 | 0.597 |
| G5 | 0.069 | 0.019 | 3.705 | <0.001* |
| HTF | 0.073 | 0.023 | 3.105 | 0.002* |

^a^ *P < 0.05.

**Supplementary Table S2**. Results of subgroup analysis (multiple linear regression analysis of the effect of embryo culture medium on birthweight z-score in the primary infertility group).

|  | β^a^ | *P*_value |
| --- | --- | --- |
| Medium (versus Cook) |  |  |
| Medium 1 (G5-PLUS) | 0.040 | 0.086 |
| Medium 2 (G5) | 0.092 | <0.001*^b^ |
| Medium 3 (HTF) | 0.094 | 0.002 |

^a^ β represents the regression coefficient.

^b^ *P < 0.05.

**Supplementary Table S3**. Results of subgroup analysis (logistic regression analysis of the effects of embryo culture medium on the risk of LGA and macrosomia in the primary infertility group).

|  | LGA |  | Macosomia |  |
| --- | --- | --- | --- | --- |
|  | OR (95%CI)^a^ | *P*_value | OR (95%CI) | *P*_value |
| Medium (versus Cook) |  |  |  |  |
| Medium 1 (G5-PLUS) | 1.16 (1.00-1.34) | 0.050 | 1.18 (0.99-1.42) | 0.075 |
| Medium 2 (G5) | 1.41 (1.21-1.63) | <0.001*^b^ | 1.35 (1.12-1.63) | 0.002* |
| Medium 3 (HTF) | 1.35 (1.12-1.62) | 0.001* | 1.26 (0.99-1.60) | 0.053 |

^a^ OR, odds ratio; 95%CI, confidence interval.

^b^ *P < 0.05.

**Supplementary Table S4**. Results of sensitivity analysis (multiple linear regression analysis of the effect of embryo culture medium on birthweight z-score after excluding embryo transfer stage from the primary model).

|  | β^a^ | *P*_value |
| --- | --- | --- |
| Medium (versus Cook) |  |  |
| Medium 1 (G5-PLUS) | 0.010 | 0.586 |
| Medium 2 (G5) | 0.071 | <0.001*^b^ |
| Medium 3 (HTF) | 0.075 | 0.001* |

^a^ β represents the regression coefficient.

^b^ *P < 0.05.

**Supplementary Table S5**. Results of sensitivity analysis (logistic regression analysis of the effects of embryo culture medium on the risk of LGA and macrosomia after excluding embryo transfer stage from the primary model).

|  | LGA |  | Macrosomia |  |  |
| --- | --- | --- | --- | --- | --- |
|  | OR (95%CI)^a^ | *P*_value | OR (95%CI) | *P*_value | |
| Medium (versus Cook) |  |  |  |  | |
| Medium 1 (G5-PLUS) | 1.06 (0.95-1.18) | 0.281 | 1.04 (0.91-1.19) | 0.610 | |
| Medium 2 (G5) | 1.25 (1.12-1.39) | <0.001*^b^ | 1.21 (1.06-1.39) | 0.006* | |
| Medium 3 (HTF) | 1.20 (1.05-1.37) | 0.007* | 1.09 (0.92-1.30) | 0.312 | |

^a^ OR, odds ratio; 95% CI, confidence interval.

^b^ *P < 0.05.

**Supplementary Table S6**. Results of sensitivity analysis (multiple linear regression analysis of the effect of embryo culture medium on birthweight z-score after excluding type of infertility from the primary model).

|  | β^a^ | *P*_value |
| --- | --- | --- |
| Medium (versus Cook) |  |  |
| Medium 1 (G5-PLUS) | 0.010 | 0.586 |
| Medium 2 (G5) | 0.071 | <0.001*^b^ |
| Medium 3 (HTF) | 0.075 | 0.001* |

^a^ β represents the regression coefficient.

^b^ *P < 0.05.

**Supplementary Table S7**. Results of sensitivity analysis (logistic regression analysis of the effects of embryo culture medium on the risk of LGA and macrosomia after excluding infertility type from the primary model).

|  | LGA |  | Macrosomia |  |
| --- | --- | --- | --- | --- |
|  | OR (95%CI)^a^ | *P*_value | OR (95%CI) | *P*_value |
| Medium (versus Cook) |  |  |  |  |
| Medium 1 (G5-PLUS) | 1.06 (0.95-1.18) | 0.281 | 1.04 (0.91-1.19) | 0.596 |
| Medium 2 (G5) | 1.25 (1.12-1.39) | <0.001*^b^ | 1.22 (1.06-1.40) | 0.006* |
| Medium 3 (HTF) | 1.20 (1.05-1.37) | 0.007* | 1.10 (0.92-1.30) | 0.300 |

^a^ OR, odds ratio; CI, confidence interval.

^b^ *P < 0.05.

**Supplementary Table S8**. Results of sensitivity analysis (multiple linear regression analysis of the effect of embryo culture medium on birthweight z-score after excluding female BMI from the primary model).

|  | β^a^ | *P*_value |
| --- | --- | --- |
| Medium (versus Cook) |  |  |
| Medium 1 (G5-PLUS) | 0.008 | 0.655 |
| Medium 2 (G5) | 0.069 | <0.001*^b^ |
| Medium 3 (HTF) | 0.069 | 0.004* |

^a^ β represents the regression coefficient.

^b^ *P < 0.05.

**Supplementary Table S9**. Results of sensitivity analysis (logistic regression analysis of the effects of embryo culture medium on the risk of LGA and macrosomia after excluding female BMI from the primary model).

|  | LGA |  | Macrosomia |  |  |
| --- | --- | --- | --- | --- | --- |
|  | OR (95%CI)^a^ | *P*_value | OR (95%CI) | *P*_value | |
| Medium (versus Cook) |  |  |  |  | |
| Medium 1 (G5-PLUS) | 1.05 (0.95-1.17) | 0.329 | 1.03 (0.90-1.18) | 0.667 | |
| Medium 2 (G5) | 1.23 (1.11-1.37) | <0.001*^b^ | 1.20 (1.05-1.38) | 0.009* | |
| Medium 3 (HTF) | 1.18 (1.03-1.34) | 0.016* | 1.07 (0.90-1.27) | 0.421 | |

^a^ OR, odds ratio; 95% CI, confidence interval.

^b^ *P < 0.05.

**Supplementary Table S10**. Results of sensitivity analysis (multiple linear regression analysis of the effect of embryo culture medium on birthweight z-score after combining the G5-PLUS group and G5 group into the Vitrolife group).

|  | β | P_value |
| --- | --- | --- |
| Medium (versus Cook) |  |  |
| Medium 1 (Vitrolife) | 0.036 | 0.030* |
| Medium 2 (HTF) | 0.069 | 0.003* |

^a^ β represents the regression coefficient.

^b^ *P < 0.05.

**Supplementary Table S11**. Results of sensitivity analysis (logistic regression analysis of the effects of embryo culture medium on the risk of LGA and macrosomia after combining the G5-PLUS group and G5 group into the Vitrolife group).

|  | LGA |  | Macrosomia |  |
| --- | --- | --- | --- | --- |
|  | OR (95%CI)^a^ | P_value | OR (95%CI) | P_value |
| Medium (versus Cook) |  |  |  |  |
| Medium 1 (Vitrolife) | 1.141 (1.036-1. 258) | 0.008*^b^ | 1.114 (0.985-1.264) | 0.088 |
| Medium 2 (HTF) | 1.184 (1.036-1.354) | 0.013* | 1.083 (0.910-1.287) | 0.369 |

^a^ OR, odds ratio; 95% CI, confidence interval.

^b^ *P < 0.05.
